# Effects of restricted compared to free arms motion during standing and walking at height in healthy adolescents

**DOI:** 10.1038/s41598-026-58618-4

**Published:** 2026-06-17

**Authors:** Anna M. Wissmann, Mathew W. Hill, Thomas Muehlbauer, Johanna Lambrich

**Affiliations:** 1 Division of Movement and Training Sciences, Biomechanics of Sport, University of Duisburg-Essen, Gladbecker Str. 182, 45141 Essen, Germany; 2 School of Psychology and Vision Sciences, University of Leicester, Leicester, UK

**Keywords:** Standing, Walking, Anxiety, Perception, Upper body strategy, Health care, Neuroscience

## Abstract

In adults, it is firmly established that height-induced postural threat as well as restriction of arms motion led to detrimental effects on emotional state and standing/walking outcomes. However, little is known about how both factors influence subjective and objective outcomes in adolescents where mechanisms to control standing and walking are still not adult-like. This work investigated emotional state and performance outcomes while standing and walking with free and restricted arms motion at ground level and at height. Twenty-five and 28 adolescents were recruited for study 1 (standing) and study 2 (walking), respectively. Participants stood (tandem stance) or walked (5 m at self-selected speed) with free or restricted arm position at both ground-level (no threat) and 80 cm above ground (threat). Postural sway (i.e., amplitude, frequency, sample entropy) and spatiotemporal gait (i.e., walking speed, time, steps, cadence) parameters were used as objective performance outcomes. Self-reported emotional state responses (i.e., balance confidence, fear of falling, perceived instability, conscious balance processing) were used as subjective indicators. In both studies, height-induced postural threat led to detrimental effects on emotional state and standing/walking outcomes. Further, adolescents in study 1 adopted a postural control strategy that differed from a “stiffening” response reported for young adults. In addition, threat-related deteriorations in spatiotemporal gait outcomes (study 2) were further amplified when arms were restricted. The findings replicate but also expand previous research about the effects of postural threat and restricted arms motion on emotional state and standing/walking outcomes and provide additional knowledge on how young people act under these conditions.

## Introduction

A significant number of studies^1–6^ have investigated the influence of height-induced postural threat on static balance tasks such as upright stance and have consistently reported a “stiffening” response. Specifically, centre of pressure (COP) amplitude decreases, while COP frequency increases when standing at height compared to standing on the ground. In addition, a recent study^1^ found that sample entropy (SampEn) increased in healthy young adults when transitioning from standing on the ground to standing above ground. This increase is indicative of enhanced ‘COP complexity’ (i.e., irregularity)^7,8^ of postural adjustments when balance is threatened. Using this non-linear balance measure—in addition to traditional parameters (i.e., COP amplitude and frequency)—provides the opportunity to better understand the motor control mechanisms (i.e., more or less adaptable postural control) underlying changes in postural sway during height-induced postural threat.

Adaptations in responses to height-induced postural threat have also been analysed for walking, revealing a decrease in walking speed and stepping accuracy (i.e., precision in hitting a foam target placed on the walkway) when walking at height compared to ground-level^9–13^. In addition to these motor adaptations, changes in psychological outcomes have also been reported. When individuals stand or walk above the ground, self-reported emotional responses indicate increased fear of falling and anxiety, a greater perceived instability and a greater conscious processing of balance^1,2,10^. However, most studies to date have focused on adults, highlighting a research gap as comparable responses in adolescents have not yet been systematically investigated. This is noteworthy, as several studies^14,15^ have reported poorer standing and walking performance in young people (children aged 3–9 years and adolescents aged 10–19 years) compared to adults. Postural control relies on the integration of sensory inputs (visual, vestibular, and somatosensory), central neural processing, and motor output to maintain balance. These systems develop progressively, with sensory integration and neural pathways undergoing significant maturation throughout childhood and adolescence, which in turn affects the ability to adapt to postural challenges^16–18^.

Researchers suggest that these mechanisms are still developing throughout childhood and adolescence^19,20^ which may affect perception, evaluation and adaptation to threat-related stimuli during standing and walking tasks. To fill the research gap, further research is needed to investigate how static (standing) and dynamic (walking) motor tasks at height—and the emotional responses they elicit—directly influence performance outcomes in adolescents. Such an investigation will allow us to better understand the extent to which the control of standing and walking is still developing in this age group.

Additional studies^2,21,22^ suggest that both emotional state and outcomes related to standing and walking at height can be positively influenced by free arms motion. Analysing the influence of arms motion during static (standing) and dynamic (walking) motor tasks offers valuable insights into compensatory strategies used to maintain stability. Specifically, analysing arms motion helps clarify their role in improving both standing and walking performance. In particular, free arm motions may act as an additional mechanism to counteract perturbations and contribute to more effective standing and walking performance, which potentially influence confidence during demanding postural tasks^2^. A recent systematic review with meta-analysis^23^ as well as original studies^2,24,25^ showed that standing with free compared to restricted arms motion resulted in a decrease in fear of falling and CoP amplitude and an increase in balance confidence and perceived stability. Similarly, in walking tasks, free arm movements have been associated with higher balance confidence and decreased step time^21^. The likely explanation for this is that restriction of arms motion limit the distribution of body mass, thereby reducing the moment of inertia, which in turn influences the stability of the postural control system^26^. While the contribution of arms motion to restoring torques and whole-body angular momentum is relatively small compared to the lower limbs^27^, they can nonetheless influence angular momentum and play an important compensatory role in postural control. During standing, free arm motions assist in maintaining postural control by adjusting limb positioning in response to balance threatening effects, especially when other sensory inputs are compromised. During walking, arm swing contributes to dynamic stability by counterbalancing the angular momentum generated by the legs. Empirical evidence shows that allowing free arms motion can help offset declines in postural control due to altered sensory conditions^25^ or increased task difficulty^24^. Therefore, it is plausible to argue that height-induced adaptations in postural control may be more pronounced when arms motion is restricted compared to when it is unrestricted. However, to date, only one study, each on performance outcomes while standing^28^ and walking^22^ has examined the role of restricted arms motion during height-induced postural threat on emotional state and standing/walking outcomes in grown-ups. Both studies^22,28^ reported response patterns in children that differed from those observed in young adults. Specifically, when standing at height, children showed a maladaptive postural strategy, i.e., an increase in COP amplitude and a decrease in COP frequency^1^, contradicting the typical “stiffening” response observed in young adults. Additionally, children compared to young adults did not reduce their cadence when walking above ground-level^22^. Again, the authors interpreted these findings as potentially less effective and maladaptive strategy to spatiotemporal gait parameters at height. Adolescents (age range: 10/12–19/20 years) are in a transition phase from childhood to adulthood^29^. For example, Steindl et al. ^16^ reported better performance while standing for adolescents compared to children, but poorer performance compared to young adults. Based on these findings, it remains unclear whether adolescents still exhibit the potentially less effective postural adaptation strategies observed in children^1,22^ or whether they already show the more adaptive and stability-protective behaviours typical of young adults^2–5^. To the best of our knowledge, this study is among the first to explore how standing or walking at height with different arm conditions (free vs. restricted arms motion) affects emotional state and standing/walking outcomes in adolescents.

Thus, the study objectives were to explore how adolescents use arm motions as a strategy to support stability under conditions of height-induced postural threat during standing and walking, and how this affects both their emotional state and standing/walking outcomes. Based on previous research^1,3,10,30–33^, it was hypothesised that height-induced postural threat would affect both emotional state and performance during standing (study 1) and walking (study 2) in adolescents, with response patterns differing from those typically observed in adults. Furthermore, we expected that these changes would be more pronounced when adolescents were unable to use their arms for corrective movements^2,21,22^.

## Materials and methods

### Participants and sample size estimation

Both studies employed a single-group repeated-measures design, investigating the within-subjects effects of postural threat (no threat vs. threat) and arm conditions (free vs. restricted) as well as the interaction thereof. Estimation of sample size for analysis of variance (ANOVA) with repeated measures was conducted by using effect sizes reported in previous studies that examined the impact of height-induced postural threat^2,9^ and/or arms motion restriction^2,34^ on standing/walking outcomes. G*Power software version 3.1.9.7^35^ was used and revealed that a minimum of 24 participants per study would be required to identify statistically significant postural threat by arm condition interactions (input parameters: effect size [*f*] = 0.25, significance level [*α*] = 0.05, power [1-*β*] = 0.80, number of groups = 1, number of measurements = 4, correlation among repeated measures *r* = .50). Consequently, 25 (study 1) and 28 (study 2) adolescents were recruited from a secondary school in Essen, North-Rhine-Westfalia, Germany (Table 1). All participants were free of any known musculoskeletal dysfunction, neurological impairment, or orthopaedic disorder. Further, the results of the visual Height Intolerance Severity Scale^36^ indicate that both samples had no relevant sign of visual height intolerance. Prior to conducting the experiment, all participants (as well as the adolescents’ parents) gave their written informed consent and the Human Ethics Committee at the University of Duisburg-Essen, Faculty of Educational Sciences approved the study protocol (approval number: EA-PSY9/24/25032024; date of approval: 25 March 2024).

**Table 1:** Characteristics of the participants by study.

Characteristic	Study 1 (standing)	Study 1 (walking)
Sample size (*n*)	25	28
Gender (females; *n*)	11	17
Chronological age (years)	14.6 ± 0.5	14.3 ± 0.6
Age range (years)	14.0–15.0	13.0–15.0
Maturity offset^1^ (years from PHV)	1.7 ± 0.7	1.6 ± 0.8
Mass (kg)	60.5 ± 13.4	58.5 ± 9.6
Height (m)	1.69 ± 0.11	1.66 ± 0.90
Body mass index (kg/m^2^)	21.0 ± 3.2	21.3 ± 3.6
vHISS	0.7 ± 1.3	1.6 ± 0.5

Note: ^1^Maturity offset (i.e., indicator of biological age) was calculated as years from PHV and classified as being either pre-pubertal (i.e., > 1 year before PHV), pubertal (i.e., ± 1 year around PHV), or post-pubertal (i.e., > 1 year after PHV). PHV, peak height velocity; vHISS, visual Height Intolerance Severity Scale, i.e., a 10-item questionnaire that provides a severity score ranging from 0 (‘Not at all’) to 13 (‘Severe’).

### Experimental procedures

**Fig. 1:**
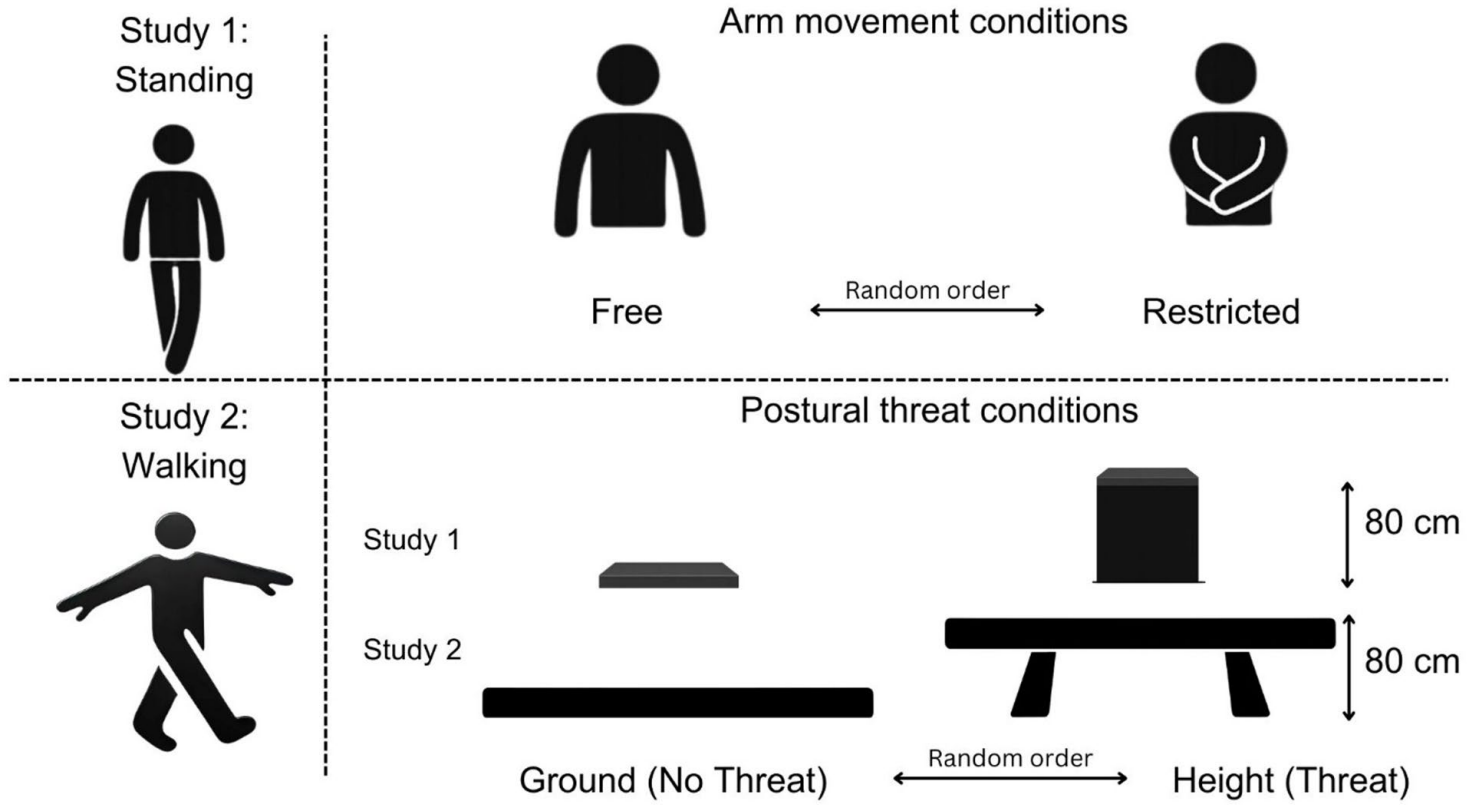
Schematic illustration of the postural threat (ground vs. height) and arm position (restricted vs. free) conditions. *Note*: The tandem stance (right foot in front of the left foot) was performed in study 1 and the 5-m walk in study 2.

Figure 1 shows the experimental protocol for both studies. Standing was investigated in study 1 (data collection period: August 2024 to October 2024) and walking in study 2 (data collection period: November 2024 to January 2025). In both studies, the assessment was conducted out under two different postural threat conditions while wearing no safety harness: (i) standing or walking at ground level (“no threat” condition), and (ii) standing or walking 80 cm above ground level (“threat” condition) on an elevated platform or walkway (commercially available balance beam [length: 500 cm, width: 10 cm], as utilized for gymnastics). The distance of 500 cm and width of 10 cm was also used for walking at ground level and was marked with adhesive tape on the floor. Access to the platform and walkway was ensured via a staircase and soft mats were placed around for safety reasons. In line with previous studies^2,3,33^, each participant performed one practice trial followed by one data-collection trial per threat condition with free (i.e., arms moved freely) and restricted (i.e., hands clasped in front of the body at waist level) arms. The order of the postural threat conditions was block randomised, as were the arm conditions. To ensure accuracy of data collection, all assessments were conducted by the same skilled assessors (graduated sport scientists).

### Assessment emotional state outcomes

The assessment of emotional state outcomes was performed in accordance with previous studies investigating young individuals^1,22^. Immediately prior to each trial, participants scored how confident they thought they feel about keeping their balance and avoiding a fall during the standing or walking task using a visual analogue scale (VAS) ranging from 0 (‘Not at all confident’) to 10 (‘Completely confident’)^30,37^. Immediately following each trial, participants rated their fear of falling during the respective task on a VAS ranging from 0 (‘Not at all fearful’) to 10 (‘Completely fearful’) ^2,4^. Participants were then asked to evaluate their perceived instability during the trial on a 0–10 VAS, where 0 correspond to being ‘Completely safe’ and 10 ‘So unsafe that I would fall’^31,38^. Additionally, participants fulfilled a questionnaire including four items to assess conscious balance processing: (1) ‘I always try to think about my balance when I perform this task’, (2) ‘I am aware of how my mind and body are functioning when performing this task’, (3) ‘I am aware of how I look when performing this task’, (4) ‘I am concerned about my movement style when performing this task’. Each item was scored on a scale from 1 (‘strongly disagree’) to 6 (‘strongly agree’) ^10^. The sum score (ranging between 4 and 24) was used for subsequent analysis, whereby a high score means a greater conscious processing of balance.

### Assessment of postural sway while standing (Study 1)

The force plate was placed on the ground in the “no threat” condition and on an elevated platform in the “threat” condition that could be reached via a staircase. Ground reaction forces and moments of forces were sampled at 1,000 Hz (AMTI AccuSway optimized, Watertown, MA, USA) and filtered (low-pass: 5 Hz) offline with a bidirectional, second-order Butterworth filter to remove high-frequency noise and measurement artefacts without affecting relevant physiological information^39^. Participants performed the tandem stance (with the left hallux touching the right posterior calcaneus) without shoes for 60 s and the trial was repeated once if stance duration was not achieved. Prior to each measurement, the force plate was calibrated by the system while no participant was standing on the plate. COP amplitude (mm), frequency (Hz), and complexity (A.u.) were assessed by calculating the root mean square (RMS), mean power frequency (MPF), and SampEn, respectively in both the anteroposterior (AP) and mediolateral (ML) directions. Reduced amplitude, coupled with increased frequency, of COP displacements reflects a postural “stiffening” response^32^. The COP time series were centred by subtracting the mean position prior to analysis. RMS and SampEn were then calculated based on the centred signals^33,40^. In contrast, MPF was derived from the power spectral density and is independent of the mean spatial position^41^. RMS was computed as the square root of the mean of the squared deviations using the following formula:

$$\:RMS=\:\sqrt{\frac{1}{N}\sum\:_{i=1}^{N}{({x}_{i}-\stackrel{-}{x})}^{2}}$$

where *x*_*i*_ is the COP value at time point *i*, $\bar x$ is the mean COP position, and *N* is the number of samples.

MPF was derived from the power spectral density (PSD), obtained via fast Fourier transform (FFT), and was calculated using the following formula:

$$\:\mathrm{MPF}=\frac{{\sum\:}_{i=1}^{N}{f}_{i}\cdot\:P\left({f}_{i}\right)}{{\sum\:}_{i=1}^{N}P\left({f}_{i}\right)}$$

where *fi* represents the frequency components and *P(fi)* the corresponding power at each frequency bin.

SampEn was computed following the approach by Richman and Moorman^42^ and with the help of the following formulas:

$$\:SampEn=\left(m,\:r,\:N\right)=\:-\mathrm{log}\left(\frac{A}{B}\right)$$

where, *m* is the length of the sequences to be compared, *r* is the tolerance value for accepting matches, *N* is the length of the data, and *A/B* are defined as follows:

$$\:A\:=\:\left\{\frac{\left(n\:-\:m\:-\:1\right)\left(n\:-\:m\right)}{2}\:\right\}\:{A}^{m}\left(r\right)$$

$$\:B\:=\:\left\{\frac{\left(n\:-\:m\:-\:1\right)\left(n\:-\:m\right)}{2}\:\right\}\:{B}^{m}\left(r\right)$$

where, $\:{A}^{m}\left(r\right)$ is the probability that sequences match for m + 1 points, and $\:{B}^{m}\left(r\right)$ is the probability that sequences match for m points. We optimised the parameter settings required for the SampEn calculation, resulting in the use of *m* = 3 and *r* = .25^43,44^.

### Assessment of spatiotemporal gait parameters while walking (Study 2)

Walking time (s) was recorded with a stopwatch to the nearest 0.01 s while participants covered the 500-cm distance (width: 10 cm) at self-selected speed wearing their own sport shoes. Further, walking speed (m/s) was calculated for each participant individually as walking distance divided by walking time and subsequently averaged across participants per test condition. Moreover, the number of steps (*n*) was counted visually and used as further outcome measure. Additionally, cadence (*n*/s) was determined by dividing the number of steps—counted from the heel contact of one foot to the heel contact of the opposite foot—by the time required to walk the 500-cm distance.

### Statistical analyses

The data were analysed using SPSS version 27.0 (IBM Inc., Chicago, IL). Assumptions of normality (Shapiro–Wilk test) and homogeneity of variance/sphericity (Mauchly test) were checked and met prior performing parametric analyses. Consistent with previous studies^1,2,10^, a series of two-way analysis of variances (ANOVAs) were performed to determine the within-subject effects of postural threat (× 2 [no threat vs. threat]) and arm condition (× 2 [free vs. restricted]). If significant threat × arm interactions were identified, *post-hoc* tests with Bonferroni-adjusted alpha levels were used to localise specific differences. Effect sizes for ANOVAs were expressed as partial eta-squared ($\:{\eta\:}_{p}^{2}$) and categorised as small (0.02 ≤ $\:{\eta\:}_{p}^{2}$ ≤ 0.12), medium (0.13 ≤ $\:{\eta\:}_{p}^{2}$ ≤ 0.25), or large ($\:{\eta\:}_{p}^{2}$ ≥ 0.26). Pairwise comparisons were indicated with Cohen’s *d* ^45^ and interpreted as trivial (0 ≤ *d* ≤ 0.19), small (0.20 ≤ *d* ≤ 0.49), moderate (0.50 ≤ *d* ≤ 0.79), or large (*d* ≥ 0.80). The alpha level for all tests was set a priori at *p* < .05.

## Results

### Study 1: Effects of restricted arms motion and postural threat on emotional state and postural sway outcomes during standing

Table 2 shows the group mean ± standard deviation values and Table 3 presents the ANOVA outputs for all assessed emotional state and postural sway outcomes during standing.

**Table 2:** Group mean ± standard deviation values for all emotional state and postural sway outcomes when standing during no threat and threat conditions with free and restricted arms motion.

Outcomes	No threat		Threat	
	Free	Restricted	Free	Restricted
*Emotional state outcomes*				
Balance confidence (0–10)	8.6 ± 1.3	8.4 ± 1.6	7.2 ± 1.6	7.0 ± 1.7
Fear of falling (0–10)	1.7 ± 1.8	2.0 ± 2.2	3.0 ± 2.2	3.6 ± 2.6
Perceived instability (0–10)	3.2 ± 2.3	3.2 ± 2.3	3.9 ± 2.2	4.7 ± 1.9
Conscious processing (4–24)	15.4 ± 3.4	15.4 ± 3.1	15.9 ± 3.5	15.6 ± 2.9
*Postural sway outcomes*				
ML-COP RMS (mm)	5.22 ± 1.21	5.33 ± 1.21	6.26 ± 1.37	6.59 ± 1.70
AP-COP RMS (mm)	8.63 ± 5.29	8.29 ± 8.16	9.37 ± 5.93	7.52 ± 3.08
ML-COP MPF (Hz)	0.50 ± 0.19	0.46 ± 0.15	0.45 ± 0.17	0.46 ± 0.14
AP-COP MPF (Hz)	0.30 ± 0.16	0.44 ± 0.28	0.35 ± 0.21	0.42 ± 0.21
ML-SampEn (A.u.)	2.46 ± 0.51	2.40 ± 0.51	2.28 ± 0.61	2.13 ± 0.53
AP-SampEn (A.u.)	2.32 ± 1.05	2.56 ± 1.08	2.21 ± 0.79	2.54 ± 0.95

Note: AP, anteroposterior; A.u., arbitrary unit; COP, centre of pressure; ML, mediolateral; MPF, mean power frequency; RMS, root mean square; SampEn, sample entropy.

**Table 3:** Main and interaction effects of the repeated measures ANOVA for emotional state and postural sway outcomes when standing.

Outcomes	Threat		Arm		Threat × arm	
	F	*p* (η_*p*_^2^)	F	*p* (η_*p*_^2^)	F	*p* (η_*p*_^2^)
*Emotional state outcomes*						
Balance confidence (0–10)	**35.552**	**< 0.001 (0.60)**	2.516	0.126 (0.10)	0.174	0.704 (0.01)
Fear of falling (0–10)	**18.254**	**< 0.001 (0.43)**	1.247	0.275 (0.05)	0.371	0.548 (0.02)
Perceived instability (0–10)	**17.790**	**< 0.001 (0.43)**	1.798	0.193 (0.07)	3.789	0.063 (0.14)
Conscious balance processing (4–24)	0.684	0.416 (0.03)	0.169	0.685 (0.01)	0.784	0.385 (0.03)
*Postural sway outcomes*						
ML-COP RMS (mm)	**41.643**	**< 0.001 (0.63)**	1.764	0.197 (0.07)	0.570	0.458 (0.02)
AP-COP RMS (mm)	0.000	0.985 (0.00)	1.755	0.198 (0.07)	0.446	0.510 (0.02)
ML-COP MPF (Hz)	0.739	0.399 (0.03)	0.332	0.570 (0.01)	1.097	0.305 (0.04)
AP-COP MPF (Hz)	0.102	0.752 (0.00)	**6.298**	**0.019 (0.21)**	0.695	0.413 (0.03)
ML-SampEn (A.u.)	**9.358**	**0.005 (0.28)**	2.909	0.101 (0.11)	0.380	0.543 (0.02)
AP-SampEn (A.u.)	0.160	0.692 (0.01)	3.287	0.082 (0.12)	0.047	0.830 (0.00)

Note: 0.02 ≤ *η*_p_^2^ ≤ 0.12 indicates small, 0.13 ≤ *η*_p_^2^ ≤ 0.25 indicates medium, and *η*_p_^2^ ≥ 0.26 indicates large effects. Bold values indicate statistically significant differences (*p* < .05). AP, anteroposterior; A.u., arbitrary unit; COP, centre of pressure; ML, mediolateral; MPF, mean power frequency; RMS, root mean square; SampEn, sample entropy.

#### Emotional state outcomes

There was a significant main effect of threat for all but one outcome (i.e., conscious balance processing), with participants reporting lower balance confidence (*p* < .001) and greater fear of falling (*p* < .001) and perceived instability (*p* < .001) during the threat condition (irrespective of arm condition). The main effect of arm and the arm × threat interaction did not reach the level of statistical significance.

#### Postural sway outcomes while standing

There was a significant main effect of threat with respect to ML-COP RMS (*p* < .001) and ML-SampEn (*p* = .005), with participants showing a larger amplitude of COP displacements and a lower ML-SampEn during the threat condition (irrespective of arm condition). Further, we observed a significant main effect of arm with respect to AP-COP MPF (*p* = .019), with participants showing a greater frequency of COP displacements during the restricted arms condition (irrespective of threat condition). The arm × threat interaction was not statistically significant.

### Study 2: Effects of restricted arms motion and postural threat on emotional state and spatiotemporal gait outcomes during walking

Table 4 illustrates the group mean ± standard deviation values and Table 5 displays the ANOVA outputs for all assessed emotional state and spatiotemporal gait outcomes during walking.

**Table 4:** Group mean ± standard deviation values for all emotional state and spatiotemporal gait outcomes when walking during no threat and threat conditions with free and restricted arms motion.

Outcomes	No threat		Threat	
	Free	Restricted	Free	Restricted
*Emotional state outcomes*				
Balance confidence (0–10)	8.6 ± 2.1	7.4 ± 2.7	8.2 ± 1.6	7.1 ± 2.4
Fear of falling (0–10)	1.9 ± 2.7	2.3 ± 2.7	2.8 ± 2.8	3.6 ± 3.2
Perceived instability (0–10)	2.2 ± 2.6	2.5 ± 2.6	2.8 ± 2.3	4.2 ± 2.7
Conscious processing (4–24)	14.9 ± 4.1	14.9 ± 3.8	15.4 ± 3.4	15.4 ± 3.9
*Spatiotemporal gait outcomes*				
Walking speed (m/s)	1.95 ± 0.51	1.76 ± 0.54	1.42 ± 0.51	1.33 ± 0.35
Walking time (s)	2.74 ± 0.76	3.14 ± 1.09	3.92 ± 1.21	4.04 ± 1.11
Step number (*n*)	5.39 ± 1.47	5.89 ± 1.66	7.21 ± 1.50	7.46 ± 1.62
Cadence (*n*/s)	1.92 ± 0.33	2.00 ± 0.42	1.94 ± 0.43	1.89 ± 0.29

**Table 5:** Main and interaction effects of the repeated measures ANOVA for emotional state and spatiotemporal gait outcomes when walking.

Outcomes	Threat		Arm		Threat × arm	
	F	*p* (η_*p*_^2^)	F	*p* (η_*p*_^2^)	F	*p* (η_*p*_^2^)
*Emotional state outcomes*						
Balance confidence (0–10)	0.575	0.455 (0.02)	**18.179**	**< 0.001 (0.40)**	0.059	0.809 (0.00)
Fear of falling (0–10)	**5.733**	**0.024 (0.18)**	3.565	0.070 (0.12)	0.462	0.503 (0.02)
Perceived instability (0–10)	**7.400**	**0.011 (0.22)**	**5.096**	**0.032 (0.16)**	2.170	0.152 (0.07)
Conscious balance processing (4–24)	1.267	0.270 (0.05)	0.002	0.964 (0.00)	0.003	0.960 (0.00)
*Spatiotemporal gait outcomes*						
Walking speed (m/s)	**28.571**	**< 0.001 (0.51)**	0.704	0.409 (0.03)	**9.491**	**0.005 (0.26)**
Walking time (s)	**28.325**	**< 0.001 (0.51)**	1.728	0.200 (0.06)	**4.910**	**0.035 (0.15)**
Step number (*n*)	**31.228**	**< 0.001 (0.54)**	0.458	0.504 (0.02)	**4.765**	**0.038 (0.15)**
Cadence (*n*/s)	0.575	0.455 (0.02)	0.122	0.730 (0.00)	1.502	0.231 (0.05)

Note: 0.02 ≤ *η*_p_^2^ ≤ 0.12 indicates small, 0.13 ≤ *η*_p_^2^ ≤ 0.25 indicates medium, and *η*_p_^2^ ≥ 0.26 indicates large effects. Bold values indicate statistically significant differences (*p* < .05).

#### Emotional state outcomes

There was a significant main effect of threat with respect to fear of falling and perceived instability, with participants reporting greater fear of falling (*p* = .024) and perceived instability (*p* = .011) during the threat condition (irrespective of arm condition). Further, we detected a significant main effect of arm for balance confidence and perceived instability, with participants reporting lower balance confidence (*p* < .001) and greater perceived instability (*p* = .032) during the restricted arms condition (irrespective of threat condition). The arm × threat interaction did not reach the level of statistical significance.

#### Spatiotemporal gait outcomes while walking

**Fig. 2:**
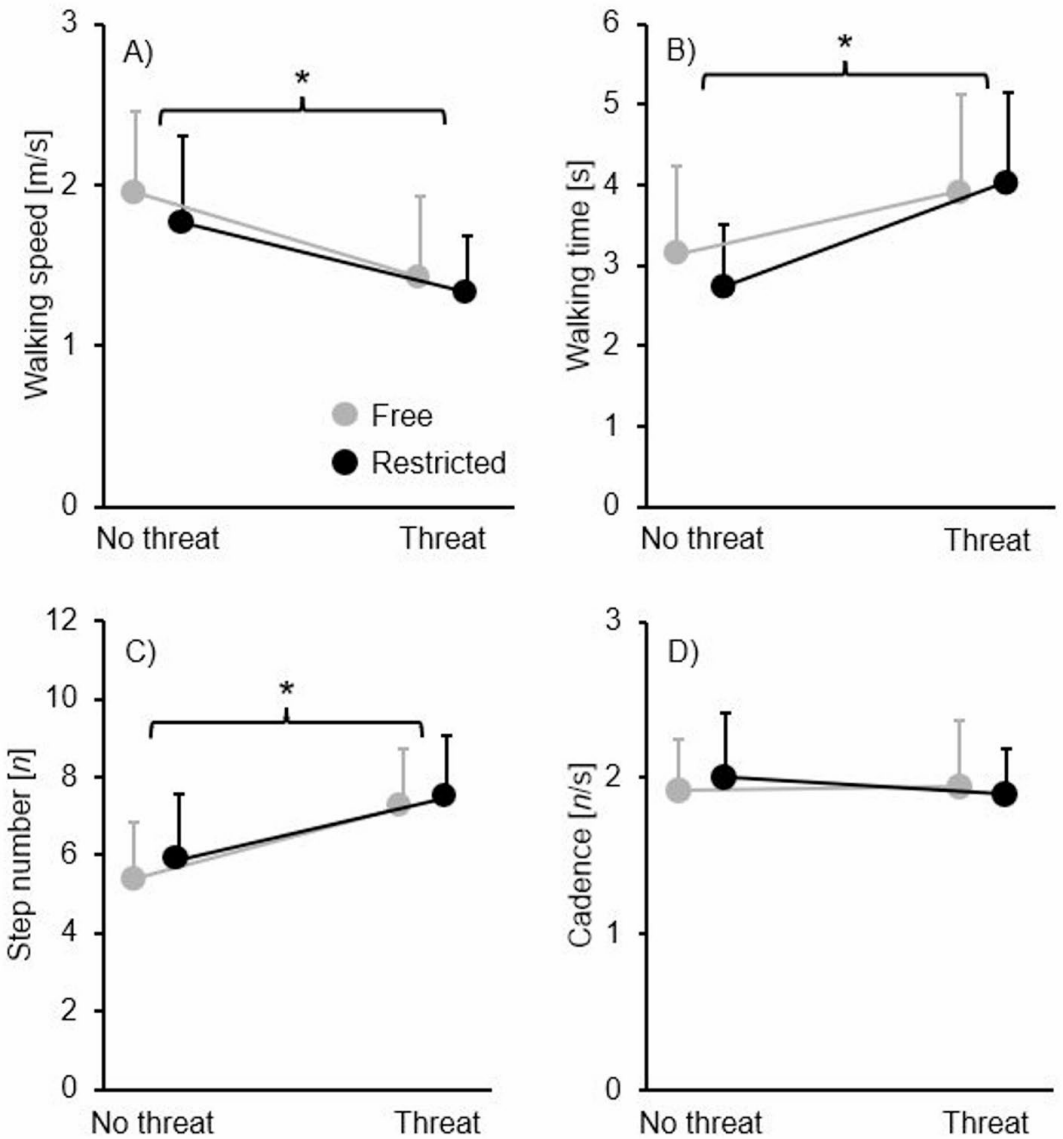
Group mean ± standard deviation values for **A**) walking speed, **B**) walking time, **C**) step number, and **D**) cadence. *Represents a significant threat × arm interaction (*p <* .05).

There was a significant main effect of threat for all outcomes (except for cadence), with participants showing lower walking speed (*p* < .001) and greater walking time (*p* < .001) and step number (*p* < .001) during the threat condition (irrespective of arm condition). Further, the arm × threat interactions were also significant for all outcomes (except for cadence). With respect to walking speed, *post-hoc* tests revealed a significant decrease from no threat to threat for both the free (*t* = 3.661, *p* < .001, *d* = 0.64) and restricted (*t* = 5.673, *p* < .001, *d* = 1.42) arms condition. Further, walking speed during the no threat condition was also significantly lower (*t* = -2.683, *p* = .006, *d* = 0.37) in the restricted compared to free arms condition (Fig. 2A). Concerning walking time, there was a significant increase from no threat to threat for both the free (*t* = -3.283, *p* = .001, *d* = 0.68) and restricted (*t* = -5.964, *p* < .001, *d* = 1.35) arms condition. Further, walking time during the no threat condition was also significantly lower (*t* = 3.073, *p* = .002, *d* = 0.38) in the restricted compared to free arms condition (Fig. 2B). In terms of step number, we observed a significant increase from no threat to threat for both the free (*t* = -3.871, *p* < .001, *d* = 0.83) and restricted (*t* = -5.817, *p* < .001, *d* = 1.34) arm conditions. Further, number of steps during the no threat condition was also significantly greater (*t* = 2.097, *p* = .023, *d* = 0.32) in the restricted compared to free arms condition (Fig. 2C). The main effect of arm was not statistically significant. Concerning cadence, neither the main effects of threat and arm nor the interaction between the two were significant (Fig. 2D).

## Discussion

This is the first study to assess emotional state and standing/walking outcomes in healthy adolescents while standing (study 1) and walking (study 2) under conditions of free and restricted arm conditions at both ground level (no threat) and height (threat). In accordance with our first hypothesis and previous literature^2,21^, standing and walking at height led to significant changes in emotional state responses. Specifically, exposure to a height-induced postural threat increased fear of falling (*η*_p_^2^ = 0.18–0.43) and perceived instability (*η*_p_^2^ = 0.22–0.43) during both standing and walking. Additionally, balance confidence (*η*_p_^2^ = 0.60) was reduced when standing above ground-level. These findings suggest that even a height of 80 cm is perceived as a postural threat, increasing adolescents perceived risk of falling. This is consistent with previous research demonstrating similar effects in healthy adults^2,3,21,46,47^. The results therefore extend earlier work by confirming that height-induced postural responses can occur even at relatively modest elevations and under the specific conditions examined in this study.

From a practical perspective, these results highlight the importance of considering height-induced fear when designing progressively challenging balance exercises for adolescents. As balance proficiency improves, gradually increasing the height of standing and walking tasks can introduce greater challenges. This structured progression may help to enhance adolescents’ perception and appropriate assessment of stable and increasingly unstable balance situations, while supporting the development of effective postural control strategies.

In addition, exposure to height-induced postural threat led to significant changes in the postural sway outcomes. Specifically, we observed an increase in COP amplitude (ML-COP RMS; *η*_p_^2^ = 0.63) and a decrease in COP complexity (ML-SampEn; *η*_p_^2^ = 0.28) when standing at height compared to standing on the ground. Both findings correspond with those of Hill et al. ^1^, who also reported significant increases/decreases in amplitude/complexity of COP movements in children. An increase in COP amplitude rather than a decrease, is contrary to the postural “stiffening” response typically reported in adults^1–5^. Similarly, the reduction in COP complexity, rather than an increase, contrasts with data reported in young adults^1^ and may reflect an alternative, maladaptive strategy for coping with height-induced postural threat. Taken together, the observed decrease in COP complexity (SampEn) alongside an increase in COP amplitude implies reduced adaptability of the postural control system, indicating that adolescents may rely on a less flexible and potentially maladaptive strategy for postural control when exposed to height-induced postural threat. This ineffective behaviour, previously only reported in children^1^, appears to persist into adolescence. As such, adolescents may struggle to adequately control threat-related stimuli, instead exhibiting a behavioural pattern more characteristic of highly fearful individuals (i.e., increased COP amplitude)^30^. A possible explanation for our observations could be the sensory^16^, neuronal^18^, and motor^17^ components of the postural control system in children and adolescents are still developing and dot not show an adult-like pattern^48^. Therefore, both from a cognitive/emotional and a motor perspective, the implementation of exercises with progressively increasing standing and walking height in adolescents seems to be an appropriate approach to promote an adult-like pattern of postural control under threatening conditions. Alternatively, Zaback et al. ^33^ demonstrated that even repeated exposure to the same height-induced postural threat led to improvements in emotional state and (some) standing/walking outcomes.

Unlike standing, we did not observe a maladaptive postural adaptation strategy when walking at height, which contradicts with one of our previous studies^22^. Specifically, Wissmann and colleagues^22^ found no changes in spatiotemporal gait outcomes (i.e., no decrease in cadence) in children compared to young adults when switching from walking on to above ground-level. In the present study, however, the change in walking height was associated with a decrease in walking speed (*η*_p_^2^ = 0.51) and an increase in walking time (*η*_p_^2^ = 0.51) and number of steps (*η*_p_^2^ = 0.54) but no significant change in cadence (*η*_p_^2^ = 0.02). These adjustments partially align with those reported for young adults^21,22^. The authors interpret these changes as evidence of a deliberate strategy to compensate for the destabilization associated with walking at height. The observation of a potentially maladaptive and ineffective postural adaptation strategy when standing at height, but not when walking, suggests that adjustments to dynamic motor task requirements develop earlier than static ones. This interpretation is supported by studies^49,50^ that reported smaller age-related differences between children, adolescents and young adults in walking versus standing tasks.

Partially in line with our second hypothesis and the literature^2,21^, restriction of arms motion amplified the threat-induced changes for parameters related to walking but not standing. Specifically, the adolescents compensated the restriction of arms motion by increasing the number of steps (*η*_p_^2^ = 0.15) and walking time (*η*_p_^2^ = 0.15), thereby reducing walking speed (*η*_p_^2^ = 0.26) even further. These more pronounced adaptations observed during walking but not standing, with restricted arm position at height indicate that control mechanisms are more demanding for walking than for standing. During standing, the base of support (i.e., the feet) and the ground (i.e., the standing surface) remain stationary, while the COP continuously oscillates around the centre of mass to maintain stability^51^. In contrast, during walking both the base of support and the centre of mass shift. This difference also results in a greater risk of falling in dynamic compared to static motor tasks^52^, which is further increased by the restriction of arms motion, which helps explain the discrepancies in the observed adaptations. For practitioners (e.g., P.E. teachers, gymnast coaches), this finding suggests that programs to promote standing/walking performance in adolescents may benefit from using changes in arm position (e.g., from arms free to move over arms akimbo to arms crossed over the chest) alongside variations in standing/walking height to enhance training progression.

While this study provides novel insights into the effects of restricted arms motion and height-induced postural threat on adolescents’ performance during standing and walking, several limitations must be acknowledged. First, the repeated measures design included a single age group only (i.e., adolescents) which limits conclusions about developmental trajectories. Longitudinal studies are needed to determine whether the observed maladaptive postural strategies persist or in which age range it changes during the transition from adolescence (≈ 13–18 years) to adulthood (> 18 years). Second, emotional state and standing/walking outcomes were analysed in healthy adolescents. Future research should extend this approach by including individuals with balance and movement disorders that are more prone to fall. Third, the use of single-trial data may reduce reliability and increase susceptibility to random variation especially for the non-linear measure SampEn. Future studies should include multiple trials per condition to assess intra-individual variability and determine the minimum important change needed to support stable conclusions. Fourth, the validity and reliability of the VAS have been evaluated for adults but not for adolescents. Although previous studies with children^1,22^ also used these scales, this fact could limit the validity of the findings. Fifth, a young adult control group was not included, which precluded direct comparisons. However, indirect comparisons with previously published findings are still appropriate, as earlier studies^2,21^ used identical motor tasks (i.e., tandem stance; walking on a wooden balance beam) and study designs (i.e., single-group repeated-measures design). Finally, the height-induced postural threat was applied at a relatively low elevation level (i.e., 80 cm). Further research should examine whether similar responses occur at greater heights or in more ecologically valid conditions, such as in sport-specific or rehabilitation settings.

## Conclusions

In conclusion, this study investigated the effects of free vs. restricted arm position on emotional state and performance parameters during standing (study 1) and walking (study 2) on ground (no threat) and 80 cm above ground-level (threat) in healthy adolescents. In both studies, height-induced postural threat led to changes in emotional state and performance parameters that differed for the upright stance from those reported for adults, indicating a potentially less effective and maladaptive strategy to control posture at height. Furthermore, the restriction of arms motion during walking at height amplified the threat-related deteriorations in spatiotemporal gait parameters, suggesting that dynamic, rather than static, motor tasks are more effective for establishing a deliberate control strategy. These findings can inform the design of training programs aimed at promoting adaptations to varying height conditions.

## Data Availability

The datasets used and/or analysed during the current study are available from the corresponding author on reasonable request.
